# Rigid Ankle Foot Orthosis Deteriorates Mediolateral Balance Control and Vertical Braking during Gait Initiation

**DOI:** 10.3389/fnhum.2017.00214

**Published:** 2017-04-28

**Authors:** Arnaud Delafontaine, Olivier Gagey, Silvia Colnaghi, Manh-Cuong Do, Jean-Louis Honeine

**Affiliations:** ^1^CIAMS, Université Paris-Sud Université Paris-Saclay, Orsay, France; CIAMS, Université d’OrléansOrléans, France; ^2^Service de Chirurgie Orthopédique, C.H.U Kremlin BicêtreKremlin Bicêtre, France; ^3^CSAM Laboratory, Department of Public Health, University of PaviaPavia, Italy

**Keywords:** ankle-foot orthosis, ankle rigidity, gait initiation, balance control, vertical braking

## Abstract

Rigid ankle-foot orthoses (AFO) are commonly used for impeding foot drop during the swing phase of gait. They also reduce pain and improve gait kinematics in patients with weakness or loss of integrity of ankle-foot complex structures due to various pathological conditions. However, this comes at the price of constraining ankle joint mobility, which might affect propulsive force generation and balance control. The present study examined the effects of wearing an AFO on biomechanical variables and electromyographic activity of tibialis anterior (TA) and soleus muscles during gait initiation (GI). Nineteen healthy adults participated in the study. They initiated gait at a self-paced speed with no ankle constraint as well as wearing an AFO on the stance leg, or bilaterally. Constraining the stance leg ankle decreased TA activity ipsilaterally during the anticipatory postural adjustment (APA) of GI, and ipsilateral soleus activity during step execution. In the sagittal plane, the decrease in the stance leg TA activity reduced the backward displacement of the center of pressure (CoP) resulting in a reduction of the forward velocity of the center of mass (CoM) measured at foot contact (FC). In the frontal plane, wearing the AFO reduced the displacement of the CoP in the direction of the swing leg during the APA phase. The mediolateral velocity of the CoM increased during single-stance prompting a larger step width to recover balance. During step execution, the CoM vertical downward velocity is normally reduced in order to lessen the impact of the swing leg with the floor and facilitates the rise of the CoM that occurs during the subsequent double-support phase. The reduction in stance leg soleus activity caused by constraining the ankle weakened the vertical braking of the CoM during step execution. This caused the absolute instantaneous vertical velocity of the CoM at FC to be greater in the constrained conditions with respect to the control condition. From a rehabilitation perspective, passively- or actively-powered assistive AFOs could correct for the reduction in muscle activity and enhance balance control during GI of patients.

## Introduction

Ankle joint plays a critical role during locomotion and is frequently prone to injury (Fuchs et al., 2003). The “traditional” ankle-foot orthoses (AFO) are rigid and designed to immobilize the ankle joint at a right angle. Such an approach is effective for preventing foot drop during swing phase ensuring toe clearance and proper contact with the heel (Yamamoto et al., 1997; Shorter et al., 2013; Alam et al., 2014). Immobilization of the ankle joint, henceforth referred to as ankle rigidity, has also been documented to reduce pain (Leung and Moseley, 2003; Thoumie et al., 2004; Richie, 2007), stimulate proprioception (Feuerbach et al., 1994; Nigg, 2001; Richie, 2007) and enhance gait for a wide range of patients suffering from severe locomotive disorders (Danielsson and Sunnerhagen, 2004; Lucareli et al., 2007; Wang et al., 2007; Brehm et al., 2008; Abe et al., 2009; Fatone et al., 2009). The current modern design of AFOs include articulated devices capable of assisting plantarflexion during stance. Whereas some studies confirmed the benefits of assistive AFOs (Guillebastre et al., 2009; Bregman et al., 2011; Eddison and Chockalingam, 2013; Petrucci et al., 2013; Kerkum et al., 2014; Kim et al., 2015), other studies have asserted a minimal effect of traditional AFOs on global gait kinematics in hemiplegic patients (Yamamoto et al., 1997; Mulroy et al., 2010). Therefore, taken into consideration the economic cost and the bulkiness of some articulated AFOs, the standard rigid model is still commonly used in rehabilitation practices.

While the effects of wearing AFOs on the general kinematic of gait during steady-state walking have been studied to a certain extent, little is known about their effects on the kinematics and EMG parameters of gait initiation (GI). GI is now a well-established experimental paradigm which has led to numerous fundamental findings. It comprises of an anticipatory postural adjustment (APA) phase and step execution phase (Carlsöö, 1966; Brenière and Do, 1986, 1991; Brenière et al., 1987). In GI, an APA has two objectives. The first is to create a disequilibrium torque in the sagittal plane which allows to initiate forward movement of the center of mass (CoM) from immobile posture. The motor strategy involves inhibition of antigravity background muscle activity of soleus (Sol) and bilateral activation of tibialis anterior (TA) which induce backward displacement of the center of pressure (CoP) relative to CoM, creating the disequilibrium torque (Crenna and Frigo, 1991; Lepers and Brenière, 1995). The magnitude of the disequilibrium torque plays a crucial role in determining global kinematic of GI (Honeine et al., 2014). The second objective of APA is to displace the CoM in the direction of the stance leg prior to step execution (Mille et al., 2014). The CoM lateral displacement towards the swing leg allows modulating the disequilibrium torque in the frontal plane to prevent a rapid medial fall and control mediolateral kinematic variables (Lyon and Day, 1997; Honeine et al., 2016; Yiou et al., 2016a,b). The CoM displacement during APA has been shown to result from loading the swing leg whilst unloading the other (Carlsöö, 1966; Winter, 1995). Loading the swing leg causes the ipsilateral movement of the CoP and contralateral movement of CoM. Honeine et al. (2016) showed that during APA, stance leg TA activity ipsilaterally flexes the knee, contributing to hip abductor activity in loading the swing leg which produces the typical displacement of CoP in the frontal plane. During step execution, stance leg Sol is activated in order to resist the action of gravity and brake the fall of the CoM (Honeine et al., 2013, 2014). Braking the fall of CoM could ease the impact of the swing limb at foot contact (FC), reducing the stress on the leg joints and providing postural stability during the subsequent double-stance phase (Kuo, 2007; Welter et al., 2007; Chong et al., 2009).

Lower leg proprioceptive afferent inputs play a major role in modulating lower leg activity during the APA phase of GI (Ruget et al., 2008, 2010; Mouchnino and Blouin, 2013). By constraining the ankle joint, an AFO would necessarily alter this somatosensory information and could thus have a deteriorating effect on motor performance during APA. Delafontaine et al. (2015) showed that ankle hypomobility induced by means of strapping the joint deteriorated both the APA and step execution phases. In addition, strapping the ankle had a tendency to impair mediolateral balance control and braking of CoM fall during single-stance.

In the present study, we investigated the effect of firm ankle rigidity caused by wearing a solid “standard” AFO on GI. We hypothesized that immobilizing the ankle should cause an ipsilateral reduction in TA dorsiflexor muscle activity during APA and Sol plantarflexor muscle activity during step execution. The reduction in TA activity during APA is expected to produce a reduction in forward and lateral CoM velocity throughout GI (Honeine et al., 2013, 2014, 2016). We also postulate that the reduction in stance leg Sol activity during step execution should impede the braking of the CoM downward fall (Honeine et al., 2013, 2014). If our hypothesis is confirmed, then this study would favor the use of articulated plantar-flexion-assisting AFOs in order to enhance dynamic balance during locomotive tasks of patients.

## Materials and Methods

### Subjects

Nineteen healthy adults (10 men and 9 women, mean age 30.3 ± 4.4 years, height 1.7 ± 0.07 m and body-mass 69.8 ± 6.2 kg) participated in this study. All subjects gave informed written consent as required by the Declaration of Helsinki. The experiment was approved by the local ethic committee of the University Paris-Saclay (EA 4532).

### Experimental Protocol

**Figure 1:**
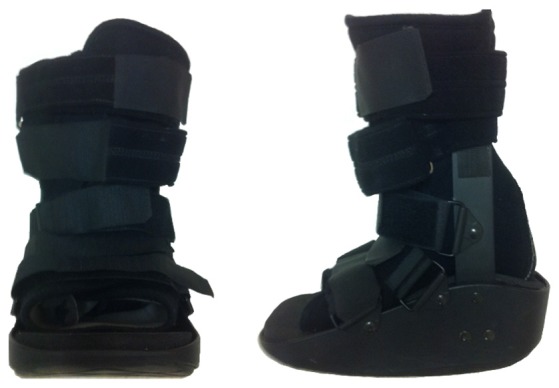
**“Standard” short ankle foot orthosis**. The figure portrays a frontal and side view of the rigid orthosis that was used in this study. The orthosis can block dorsi- and plantarflexion of the ankle in addition to reducing the eversion and inversion of the foot.

Subjects stood on a force platform (0.9 × 1.80 m, AMTI, Watertown, MA, USA). They were asked to initiate gait at a self-paced speed following an acoustic signal. Subjects were specifically instructed not to start walking in a reaction-time mode, but to start when they felt ready (this usually occurred following an interval of 0.5–1 s). They performed GI under three experimental conditions: GI without wearing an orthosis (Ctrl), GI while wearing the orthosis on the stance leg ankle (O-St), and GI while wearing the orthoses on both ankles (O-Bi). The order of the conditions was randomly assigned. Before recording, preferential starting leg of the subjects was established. Subjects were asked to stand still eyes closed, and a small thrust was applied to their back forcing them to make a step forward. This was repeated three times. Subjects were instructed to initiate gait with the stepping leg that was used during this test. Each experimental condition comprised 10 trials. Biomechanical variables, obtained from each trial, were calculated (see below). The mean of the 10 trials for each variable was then computed. Subjects were asked to wear everyday sneaker shoes. In the O-St conditions, subjects kept wearing the shoe on the swing side in order to match the elevation of the orthosis and mimic real life situation. A rigid “standard” short ankle foot orthosis (“botte de marche courte MaxTrax^®^ Ankle”, Donjoy^®^) was used in this study (Figure 1). The orthosis was designed to prevent plantar/dorsi flexion and eversion/inversion movements of the ankle (Thoumie et al., 2004).

### Acquisition and Measurements

Ground reaction forces and CoP data were obtained from the force platform. Surface EMG activity of TA and Sol was recorded using bipolar Ag-AgCl electrodes via wireless preamplifiers (Zero-wire, Aurion, Milan, Italy). Electrode sites and preparation was performed according to the SENIAM protocol (Merletti and Hermens, 2000). EMG raw traces were bandpass filtered (10–500 Hz) with a second order Butterworth no-lag filter. Force platform and EMG data were digitized with an analog to digital converter at a sampling frequency of 1000 Hz and saved on a PC for off-line analysis.

**Figure 2:**
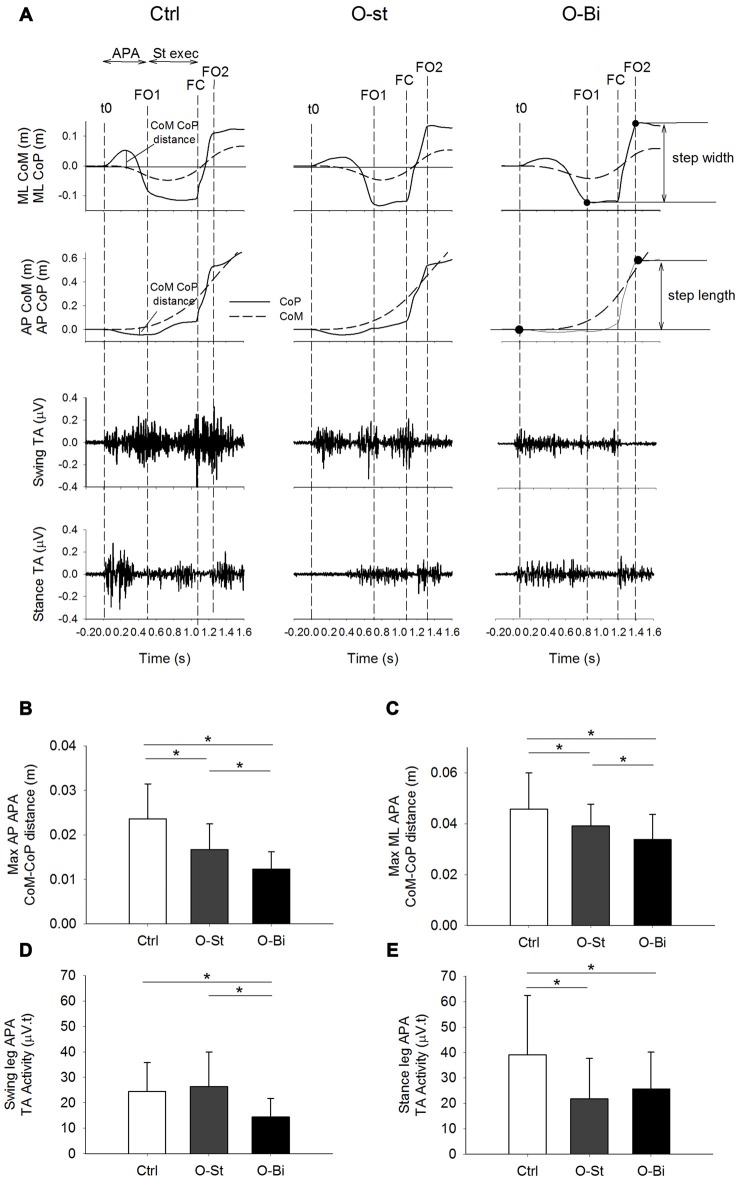
**Anteroposterior (AP) and mediolateral (ML) center of mass (CoM)-center of pressure (CoP) distance and the bilateral tibialis anterior (TA) activity**. Panel **(A)** shows, from top to bottom the timelines of the ML and AP CoP (solid lines) and CoM (dashed lines) trajectories in addition of the raw traces of swing and stance TA activity during gait initiation (GI). The traces were obtained from a single trial of a representative subject in the Ctrl (left), O-St (middle) and O-Bi (right) conditions. The vertical dash lines represent the instant of *t*0, first foot off (FO1), foot contact (FC) second foot off (FO2). Panels **(B–E)** show the grand means (*N* = 19) and standard deviations of the maximum AP and ML distance between the CoM and CoP, the swing and stance TA activity. The histograms show that wearing the ankle-foot orthoses (AFO) decreases the activity of TA and ML and AP CoP and CoM excursions. *Indicates significant difference (*p* < 0.05).

The mediolateral (ML) CoP instantaneous position curve was used to determine the onset of GI (*t*0), first foot off (FO1) and FC, in addition to the second foot off (FO2; Figure 2). The instant of *t*0 was determined as the instant when the ML CoP trace deviated 2 standard deviations from its baseline value. The moments of foot offs and that of FC were determined as the local minimums of the second derivative of the ML CoP trace. Visual inspection was conducted on all trials to verify the correctness of the algorithm.

**Figure 3:**
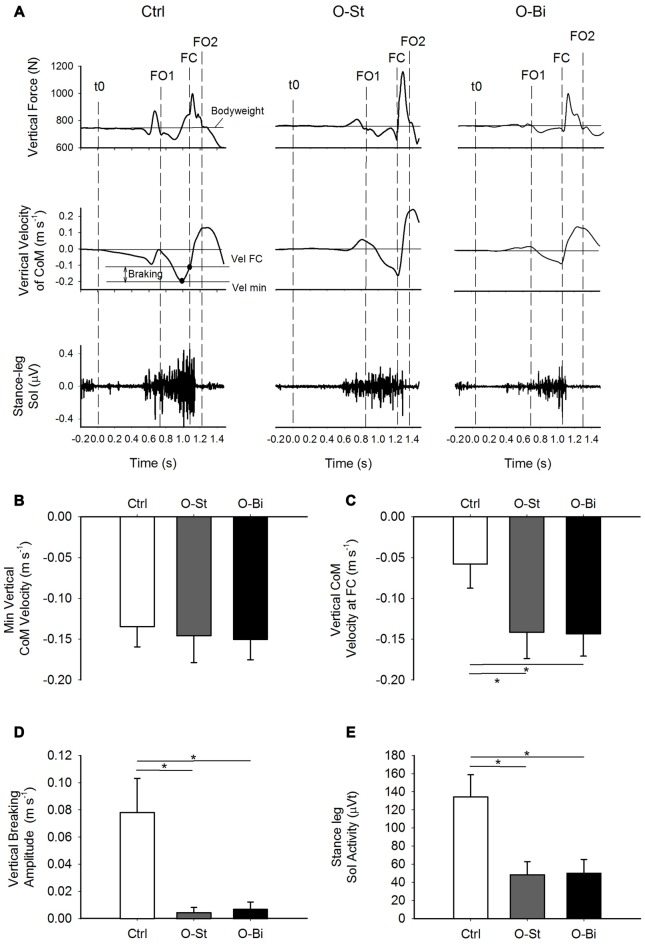
**Vertical ground reaction force and CoM velocity and the stance leg Sol activity**. Panel **(A)** shows, from top to bottom) the timelines of the vertical ground reaction forces and vertical CoM velocity in addition of the raw traces of stance Sol activity during GI. The traces were obtained from a single trial of a representative subject in the Ctrl (left), O-St (middle) and O-Bi (right) conditions. The vertical dash lines represent the instant of *t*0, first foot off (FO1), FC second foot off (FO2). Panels **(B–E)** show the grand means (*N* = 19) and standard deviations of the minimum vertical velocity of CoM, the vertical velocity of CoM at FC, the vertical braking of CoM vertical fall and the stance Sol activity during step execution. It can be noted that wearing the AFO decreases the activity of Sol, which affects the braking action on the CoM vertical fall. *Indicates significant difference (*p* < 0.05).

The APA phase was considered to be the time-window spanning from *t*0 until FO1. The step execution phase was considered as the period between foot off and FC. Step length was approximated as the distance between the anteroposterior (AP) position of CoP at the instants of *t*0 and the FO2 (Figure 2). Step width was considered to be the distance between the ML position of the CoP at the instants of the FO1 and FO2 (Figure 2). The CoM acceleration in the ML and AP directions were calculated by dividing the respective ground reaction forces by the subjects’ mass. The CoM vertical acceleration was obtained by subtracting the subjects’ bodyweight from the vertical ground reaction force and dividing by the subjects mass. The CoM velocity in all three directions was then obtained by integrating the respective acceleration with respect to time. The magnitude of the vertical braking during step execution was measured as the difference between the minimum vertical velocity of CoM during single support and its vertical velocity at FC (Figure 3).

EMG raw traces were rectified and then low-pass filtered at a cut-off frequency of 25 Hz with a no-lag second order Butterworth filter. Amplitudes of EMG activity of each muscle were calculated by integrating the respective EMG filtered trace. Amplitudes of TA activity of both legs were calculated from the moment of onset until the instant of foot off. Amplitudes of stance leg Sol activity were calculated from the moment of onset until the instant of FC. The moment of muscle onset was calculated using a custom-made algorithm based on continuous (Morlet) wavelets transform (see Honeine et al., 2016).

### Statistical Analyses

The Shapiro-Wilk test was used to determine if the studied variables were normally distributed. If ShapiroWilk test was significant (i.e., SW-*p* < 0.05), then the hypothesis that the data is normally distributed should be excluded. In the result section, the normality distribution tests are presented in the following condition order: Ctrl, O-St and O-Bi. Repeated-measures analysis of variances (ANOVAs) were used to test the effect of the three experimental conditions on the kinematics and EMG parameters. A significant outcome was followed up with the Bonferroni correction *post hoc* test. The threshold of significance was set at *p* < 0.05.

## Results

We first analyzed general kinematic variables of GI. Table 1 contains the grand mean and standard deviation of the duration of APA and step execution. Wearing the AFO, i.e., in the O-St and O-Bi conditions, had a significant effect on the durations of both the APA and step execution phases (SW-*p*: 0.16, 0.44, 0.22—*F*_(2,36)_ = 38, *p* < 0.001; SW-*p*: 0.24, 0.11, 0.82—*F*_(2,36)_ = 55, *p* < 0.001, respectively). *Post hoc* analyses showed that wearing the orthosis increased the duration of APA, more so when the AFO was worn on the stance leg than bilaterally (*p* < 0.001 for both comparisons). The duration of step execution decreased only in the O-St condition (*p* < 0.001).

**Table 1:** **Grand means (*N* = 19) and standard deviations of the durations of gait initiation (GI) phases**.

	Duration of APA (s)	Duration of step execution (s)
Ctrl	0.62 ± 0.04	0.37 ± 0.02
O-St	0.74 ± 0.05*	0.31 ± 0.03*
O-Bi	0.67 ± 0.05^*◊^	0.38 ± 0.03^◊^

*Significantly different than Ctrl, ^◊^significantly different than O-St.

### APA in the Anteroposterior Direction

The time-profiles of the CoM (dashed line) and CoP (solid line) during the first step in the sagittal plane in addition to the TA activity of both legs are presented in Figure 2A top panels. The traces were obtained from one subject during all three conditions: Ctrl (left), O-St (middle) and O-Bi (left). As seen in the figure, CoP is displaced backwards during APA. The displacement is accompanied by bilateral activation of the TA muscles. Grand mean and standard deviation of the maximal distance between CoM and CoP during APA in AP direction in addition to the amplitude of EMG activity of both TA muscles are shown in the Figures 2B,D,E.

ANOVA showed a significant effect of wearing the orthosis on the maximum CoM-CoP distance in the AP direction during the APA phase (SW-*p* for the Ctrl, O-St and O-Bi respectively: 0.95, 0.18, 0.24—*F*_(2,36)_ = 30.3, *p* < 0.001). *Post hoc* analyses showed that the maximum AP CoM-CoP distances observed during APA were greatest in the Ctrl condition and smallest in the O-Bi condition (*p* < 0.05 for all comparisons). ANOVA also showed an effect of wearing the orthosis on the activity of TA (swing leg: SW-*p*: 0.67, 0.72, 0.15—*F*_(2,36)_ = 18.7, *p* < 0.001; stance leg: SW-*p*: 0.14, 0.31, 0.38—*F*_(2,36)_ = 24.4, *p* < 0.001). *Post hoc* analyses showed that the activity of TA was always smaller when the ankles were constrained by the orthosis with respect to not wearing it (*p* < 0.001 for all comparisons).

### APA in the Mediolateral Direction

Figure 2A also portrays the displacement time profiles of the CoM (dashed line) and CoP (solid line) in the frontal plane in all three conditions. During APA, CoP is displaced laterally in the direction of the swing foot. The displacement of CoP causes a movement of the CoM in the opposite direction. ANOVA showed a significant effect of wearing the orthosis on the maximum ML CoM-CoP distance during the APA phase (SW-*p*: 0.16, 0.4, 0.73—*F*_(2,36)_ = 21.6, *p* < 0.001, respectively). *Post hoc* analyses showed that the maximum ML CoM-CoP distances measured during APA were greatest in the Ctrl condition and smallest in the O-Bi condition (*p* < 0.05 for all comparisons). Grand means and standard deviations are shown in Figure 2C.

### Kinematic Variables of Step Execution

Grand means and standard deviations of the AP and ML velocity of CoM measured at FC, as well as step length, and step width are shown in Table 2. Wearing the orthosis significantly changed the instantaneous AP velocity measured at the instant of FC (SW-*p*: 0.19, 0.75, 0.28—*F*_(2,36)_ = 37.9, *p* < 0.001). *Post hoc* tests revealed that the AP velocity of the CoM decreased in both the O-St and O-Bi conditions (*p* < 0.001 in both comparisons). However, the AP CoM velocity was higher in the O-Bi condition with respect to O-St (*p* < 0.05). The orthosis did not modify step length (SW-*p*: 0.46, 0.21, 0.11—*F*_(2,36)_ = 0.32, *p* = 0.74). Wearing the rigid AFO also changed the ML velocity of CoM measured at FC (SW-*p*: 0.07, 0.12, 0.39—*F*_(2,36)_ = 60.3, *p* < 0.001). *Post hoc* analyses showed an increase in ML velocity of the CoM, more so when it was applied bilaterally than to the stance leg alone (*p* < 0.05 for all comparisons). Step width was also significantly affected as a result of wearing the AFO (SW-*p*: 0.47, 0.69, 0.67—*F*_(2,36)_ = 55, *p* < 0.001). *Post hoc* analyses showed that step width increased in O-St with respect to Ctrl and was largest in the O-Bi condition (*p* < 0.01 for both comparisons).

**Table 2:** **Grand means (*N* = 19) and standard deviations of general kinematics variables of GI**.

	AP velocity at foot contact (m/s)	ML velocity at foot contact (m/s)	Step length (m)	Step width (m)
Ctrl	1.05 ± 0.10	0.16 ± 0.05	0.55 ± 0.04	0.17 ± 0.04
O-St	0.87 ± 0.11*	0.19 ± 0.06*	0.53 ± 0.05	0.21 ± 0.05*
O-Bi	0.912 ± 0.12^*◊^	0.23 ± 0.06^*◊^	0.55 ± 0.07	0.24 ± 0.05^*◊^

*Significantly different than Ctrl, ^◊^significantly different than O-St.

### Active Vertical Braking during Step Execution

The time-*p*rofiles of the vertical ground reaction force, the CoM vertical velocity curves (upper panels) and of the EMG activity of the stance leg soleus (lower panels) obtained from one trial of a single subject in the Ctrl (left), O-St (middle) and O-Bi (left) conditions are shown in panel A of Figure 3. As can be seen in the figure, following foot off, the CoM accelerated downward (negative velocity indicates downward movement of CoM) and then reversed. In fact, during single support, the CoM velocity shows a “V” shape indicating that the CoM fall was braked. In Ctrl condition, the braking action, which is accompanied by a surge in stance soleus activity, caused a reduction in the absolute of vertical velocity of the CoM measured at FC. In the O-St and O-Bi conditions, the soleus activity of stance leg was reduced and minimum absolute velocity at FC, in most cases, was recorded at FC. This reveals that constraining the stance leg with a rigid AFO has a deteriorating effect on the active vertical braking that occurs during unconstrained GI.

Grand means and standard deviations of the minimum vertical velocity of CoM during single-support, the vertical velocity of CoM measured at the instant of FC, the active vertical braking of CoM and the amplitude of the stance leg Sol activity during single-support are shown in Figure 3 (lower panels). ANOVA showed no effect of the orthosis on the absolute minimum vertical velocity of CoM during single-support across the conditions (SW-*p*: 0.34, 0.52, 0.14—*F*_(2,36)_ = 0.28, *p* = 0.54). However, wearing the orthosis had an effect on the vertical velocity of CoM measured at the instant of FC (SW-*p*: 0.12, 0.6, 0.19—*F*_(2,36)_ = 106.7, *p* < 0.001) and the magnitude of vertical braking of CoM during single-stance (SW-*p*: 0.52, 0.57, 0.07—*F*_(2,36)_ = 266.1, *p* < 0.001). *Post hoc* analyses revealed that both the absolute vertical velocity of CoM at FC and the amplitude of vertical braking of CoM were significantly smaller in the O-St and O-Bi condition with respect to the Ctrl conditions (*p* < 0.001). *Post hoc* analyses showed that the absolute vertical minimum velocity of CoM and the absolute velocity at FC measured in O-St and O-Bi conditions were comparable (*p* > 0.05). Wearing the orthosis also had an effect on the amplitude of the stance leg Sol activity during single-support (SW-*p*: 0.18, 0.31, 0.26—*F*_(2,36)_ = 101.4, *p* < 0.001). *Post hoc* analyses showed that the amplitude of the stance leg Sol activity was lowest in the O-St and O-Bi (*p* < 0.001).

## Discussion

The results of the present study show that AFOs cause an ipsilateral reduction in TA activity during APA, and ipsilateral decrease of Sol activity during step execution. The decrease in muscle activity is accompanied by a decrease in AP CoM velocity and an increase in ML CoM velocity. In addition, constraining the stance leg ankle joint reduced the vertical braking of the CoM fall that is observed in the single-stance phase of normal GI.

Foot and ankle proprioceptive inputs are known to play a role in modulating lower leg activity during the APA phase of GI (Ruget et al., 2008, 2010; Mouchnino and Blouin, 2013). The modification of lower limb muscle activity in this study may be linked to alteration in the proprioceptive foot and ankle inputs that are caused by constraining the ankle with a rigid AFO. In line with Delafontaine et al. (2015), the reduction in AP velocity measured at FC, with respect to control, is greater in the O-St than in the O-Bi condition. Delafontaine et al. (2015) suggested that the higher AP CoM velocity in the double-constrained condition is probably due to the better capacity of the brain to deal with a symmetrical change of proprioceptive inputs, as opposed to the asymmetrical somatosensory modification that occurs when only one ankle is constrained. The increase in AP velocity in the O-Bi condition could also be caused by adjusting trunk position in order to advance the position of the CoM relative to the base of support and increase forward momentum, which is thought to occur in lower limb amputees (Michel and Do, 2002).

Our evidence that induced stance leg ankle rigidity reduces the activity of the TA is consistent with the results of Geboers et al. (2002). During the APA phase of GI, the bilateral increase in TA activity accompanied by the silencing of both Sol muscles is responsible for generating a forward momentum (Crenna and Frigo, 1991; Honeine et al., 2013, 2014). It may be noted that the decrease in TA activity was compensated by an increase in the duration of APA in order to allow the gravitational torque to accumulate more pace and reach higher forward velocity. Nonetheless, the forward velocity reached at FC when the stance leg was constrained is lower than in the control condition. In addition, Honeine et al. (2016) have shown that TA activity during APA is greater in the stance leg than the contralateral limb. This causes a slight stance leg knee flexion which assists the hip abductor activity in loading the future swing leg and displacing the CoP in the direction of the swinging leg (Carlsöö, 1966; Winter, 1995). In the present study, reduction of the activity of stance TA activity also results in a decrease in ML CoP displacement during APA, corroborating the results of Honeine et al. (2016). In line with Caderby et al. (2014) and Honeine et al. (2016), for the same initial stance width, a smaller CoP displacement during APA causes the ML distance between the CoM and CoP to be larger during the subsequent step execution. This produces a larger gravitational torque during the single-support phase. As a result, the velocity of the medial fall during single-stance increases, prompting a rise in step width to restore stability in the frontal plane.

The results of this study also show that wearing the AFO on the stance leg (i.e., in the O-St and O-Bi condition) reduces the activity of the stance leg soleus EMG during single-support, as shown in studies investigating steady-state walking (Yamamoto et al., 1993; Boninger and Leonard, 1996; Miyazaki et al., 1997; Akizuki et al., 2001). In normal gait, during the single-stance phase, the body rotates around the ankle-forefoot articulation system causing the CoM to accelerate downwards. In healthy individuals, the CoM downward velocity is reduced prior to FC (Chong and Do, 2003). This active braking of CoM during single-stance is the result of triceps-surae activity (Honeine et al., 2013, 2014). In our study, the decrease of the stance leg soleus activity reduces the effectiveness of vertical braking of the CoM fall during the step-execution phase and substantially increases the shock between the swing leg and the ground at FC. In other words, the downward fall of the CoM is halted mechanically by the impact of the swing foot with the ground. On the one hand, Chong and Do (2003) and Welter et al. (2007) state that the main aim of the active braking is to reduce the shock between the heel of the swing leg and the ground. On the other hand, the dynamic inverted pendulum model provided by Kuo (2007) suggest that the vertical braking action observed in late single-stance is required to minimize the work that is necessary to lift the CoM during the subsequent double-support phase. Hence, the triceps surae braking action is necessary for the proper execution of the step-to-step transition.

Furthermore, it should be kept in mind that AFOs are traditionally designed with the main objective of preventing foot-drop in order to allow for toe clearance and promote contacting the floor with the heel instead of the metatarsals (Yamamoto et al., 1997; Shorter et al., 2013; Alam et al., 2014). Nonetheless, many “modern” AFOs have been designed in order to assist plantarflexion. Such devices can be broadly classified into two categories: passive and active devices. Passive AFOs generally employ spring mechanisms in order to store energy during single-stance, later releasing it in order to assist plantarflexion. The types of springs in passive AFO vary from mechanical (Guillebastre et al., 2009), pneumatic (Ferris et al., 2005), carbon composites material (Zou et al., 2014), oil damper (Ohata et al., 2011), and magneto reological damper (Svensson and Holmberg, 2008). Active AFOs operate an actuator in order to perform a torque across the ankle joint. Most common actuators are small electric motors (Bai et al., 2015) or pneumatic pumps (Chin et al., 2009). Furthermore, some actively-powered AFOs are designed to be controlled through EMG activity (Ferris et al., 2005; Cain et al., 2007; Wentink et al., 2013). Such a control system is thought to increase the efficiency of the AFO by enhancing the timing during which the assistive torque is generated (Alam et al., 2014).

Assistive AFOs have been shown to enhance push-off which restores active braking during late single-stance. The effect of those assistive AFOs have been shown to be beneficial for cerebral palsy patients (Eddison and Chockalingam, 2013; Kerkum et al., 2014) and hemiplegic patients following a stroke (Kim et al., 2015). In addition, Bregman et al. (2011) have shown that wearing a spring-assisted AFO decreases the energy cost of gait in stroke patients by about 10%. Their result corroborates with the hypothesis of Kuo (2007) who states that vertical force applied during late single-stance helps to reduce the energetic cost required to raise the CoM during double stance. In addition, Petrucci et al. (2013) showed that mechanically-generating plantarflexion across the swing leg ankle during APA increases the mediolateral displacement of the CoP. It is important to note that the negative effects that were induced by the rigid AFO are comparable to those described in impaired gait consecutive to different diseases. For instance, in hemiparetic stroke subjects, the preparatory ML displacement of CoP is reduced when gait is initiated with the affected limb (Hesse et al., 1997). In addition, progressive supranuclear palsy patients (Welter et al., 2007), parkinsonians (Chastan et al., 2009a,b) and elderly subjects (Chong et al., 2009) have been already documented to have deficits in braking the downward acceleration of the CoM during single-stance. It is thought that such locomotive symptoms are due to supraspinal complications affecting the central command responsible for the proper generation of APA (Rocchi et al., 2012) and step execution (Demain et al., 2014). In this study, a population of healthy young subjects was tested and the effects were obtained by mechanically constraining the ankle joint. Based on our data alone it is not possible to state whether constraining the stance leg further deteriorates the central command of patients which could worsen balance control and the active braking of gait. For instance, Chen et al. (2015) have shown that wearing an anterior (flexible) AFO improves lateral CoM displacement in stroke patients during upright stance. Further research should be performed in order to investigate whether rigid AFO could have an increased negative effect on patients and whether assistive AFO could restore balance control and active braking.

### Limitations

Wearing AFO enlarges the area of the base of support of the subjects. In addition, the same AFO was used regardless of the subjects’ shoe size. Our present data do not allow to differentiate between the influence of the enlargement of the base of support and that of constraining the ankle. In addition, wearing the AFO also increases the volume of the whole shank. Therefore, it could be possible that the increase in step-width in the O-St and O-Bi conditions is a precautious strategy aimed at preventing the ankle to bump into the AFO or a collision between the two AFOs during the execution of the second step. Future experiments including a variation of initial feet-width and/or wearing slimmer AFOs should untangle this problem. In impaired locomotive neurological patients such as Parkinsonians, this technical problem could have two ambivalent effects. Indeed, it could further deteriorate balance control or improve it, as subjects should walk with enlarged step-width. Future experiments should shed light on this question.

## Conclusions

Blocking ankle movement or limiting it disturbs kinematics gait parameters, balance control in the frontal plane and deteriorates the vertical braking action during single-stance. It would be interesting to test whether utilization of assistive AFO devices could permit to regain normal or nearly normal gait, i.e., walking with normal equilibrium and without ankle pain.

## Author Contributions

AD contributed with project creation, data collection, data analysis and drafted the manuscript. OG contributed with project creation, data analysis. SC contributed with project creation, data analysis. M-CD and J-LH contributed with project creation, data collection, data analysis. All authors discussed the results and participated in the revision of the manuscript.

## Funding

This study was supported in part by the grant “Projet Attractivité” from the Paris-Sud University.

## Conflict of Interest Statement

The authors declare that the research was conducted in the absence of any commercial or financial relationships that could be construed as a potential conflict of interest.
